# High Complete Response Rate in Patients With Metastatic Renal Cell Carcinoma Receiving Autologous Cytokine-Induced Killer Cell Therapy Plus Anti-Programmed Death-1 Agent: A Single-Center Study

**DOI:** 10.3389/fimmu.2021.779248

**Published:** 2022-01-04

**Authors:** Lingdi Zhao, Tiepeng Li, Yongping Song, Yonghao Yang, Baozhen Ma, Yong Zhang, Yiman Shang, Benling Xu, Jindong Guo, Peng Qin, Lu Han, Xiaomin Fu, Hongwei Lin, Liang Liu, Xiubao Ren, Zibing Wang, Quanli Gao

**Affiliations:** ^1^ Immunotherapy Department, Affiliated Cancer Hospital of Zhengzhou University & Henan Cancer Hospital, Zhengzhou, China; ^2^ Hematology Department, Affiliated Cancer Hospital of Zhengzhou University & Henan Cancer Hospital, Zhengzhou, China; ^3^ Biotherapy Department, Tianjin Medical University Cancer Institute and Hospital, Tianjin, China

**Keywords:** complete response, cytokine-induced killer cell, metastatic renal cell carcinoma, nivolumab, pembrolizumab, immunotherapy

## Abstract

**Background and Objective:**

The results of the CheckMate 025 trial established the status of nivolumab in the second-line treatment of metastatic renal cell carcinoma (mRCC), with an objective response rate (ORR) of 25% and a complete response (CR) rate of 1%. Thus, the efficacy of anti-programmed death (PD)-1 antibodies in the second-line treatment of mRCC requires improvement. The purpose of this study was to explore the clinical efficacy and safety of anti-PD-1 agents combined with cytokine-induced killer (CIK) cell therapy for refractory mRCC.

**Patients and Methods:**

Patients with mRCC refractory to previous targeted therapy were included in this study. All patients received anti-PD-1 plus CIK cell therapy. The ORR and CR rate, progression-free survival (PFS), overall survival (OS), and safety were assessed.

**Results:**

CR was observed in seven of the 29 patients, and partial response was observed in five patients. The ORR was 41.4% and the median PFS was 15.0 months. Up to the last follow-up, 15 patients died with an average survival time of 37 months. Among the patients who achieved a CR, one experienced cerebellar metastasis 18.8 months after discontinuation, but achieved CR again after localized gamma knife and 1-month axitinib treatment. This regimen was tolerated well and there was no treatment-related death.

**Conclusions:**

Combination therapy with anti-PD-1 and CIK cell therapy is safe and effective in patients with mRCC refractory to previous targeted therapy. The high CR rate and long disease-free survival even after long-term discontinued therapy suggest that this combination treatment may represent a potential curative regimen for this type of malignancy.

## Introduction

Renal cell carcinoma (RCC) is one of the common malignant tumors of the urinary system. In the past 20 years, the incidence of RCC has increased significantly in China, especially in urban areas (1). During this time, there has been a shift in the treatment for metastatic RCC (mRCC) from traditional non-specific cytokine therapy to targeted therapy with antivascular endothelial growth factor (VEGF)/vascular endothelial growth factor receptor (VEGFR) agents, with newly developed immunotherapeutic agents being used more recently (2, 3). Anti-VEGFR therapy upregulates the expression of programmed death-ligand 1 (PD-L1) in cancer cells, thus rendering PD-L1 a potential target and driver of resistance to antiangiogenic therapy (4). PD-L1 expression in cancer cells can induce programmed death (PD)-1 expression on T cells, thus hindering their antitumor potential through PD-1/PD-L1 signaling. Nonetheless, anti-PD-1 therapy is only effective when T cells are present in the cancer microenvironment (5–7). Despite the robust effectiveness of anti-VEGFR agents against mRCC, some tumors are inherently resistant to this treatment, and most acquire resistance over time, thereby limiting the durable clinical benefit (8). Subsequent therapy for such targeted treatment-refractory patients remains a critical issue, warranting new regimens with different mechanisms of action. The CheckMate 025 trial introduced nivolumab monotherapy as second-line therapy for mRCC, reporting an objective response rate (ORR) of 25% and a complete response (CR) rate of 1% (9). The CheckMate 025 study was conducted mainly in Europe and the United States; although approximately 10% of the included patients were of Asian ethnicity, the results of the Asian subgroup were not specifically reported. Therefore, it is necessary to explore the efficacy of anti-PD-1 antibody in the Asian population.

Cytokine-induced killer (CIK) cells exhibit non-major histocompatibility complex (MHC)-restrictive antitumor potential. Adoptive CIK cell therapy is reportedly safe and effective for mRCC, with an ORR of 15%–50% (10, 11). Transfused CIK cells can selectively infiltrate tumors, generate a local inflammatory microenvironment (12, 13), and enhance the efficacy of anti-PD-1 therapy, which disrupts the inhibitory signals of the tumor microenvironment and activates T cells. Co-induction with anti-PD-1 plus anti-CTLA-4 antibodies has been suggested to improve the antitumor activity of dendritic cell (DC)–CIK cells by promoting their proliferation, differentiation, and early activation and regulating the secretion of immune-stimulatory cytokines in carcinoma cell lines (14). Another study showed that the combination of immune checkpoint inhibition with CIK cells augmented the cytotoxicity of CIK cells against renal cancer cells by increasing interferon-γ secretion (15). Other studies reported that the expression of PD-1 on CD3^+^ CIK cells was 3.9% ± 0.5%, whereas the expression of PD-L1 on lymphoma cells increased significantly after co-culture with CIK cells (15, 16). A retrospective study in patients with advanced non-small cell lung cancer showed that the level of CD3^+^CD56^+^CD16^+^ T cells in the peripheral blood significantly increased after anti-PD-1 antibody plus CIK cell therapy, whereas the level of myeloid-derived suppressor cells (MDSC) did not change significantly; by contrast, there was no change in the level of CD3^+^CD56^+^CD16^+^ T cells in the peripheral blood for patients who received anti-PD-1 monotherapy although the MDSC level increased significantly (17).

CIK cells might modulate the tumor microenvironment by reducing the level of MDSC in peripheral blood (11). Therefore, the combinatorial administration of CIK cells and anti-PD-1 agents may have a synergistic effect in theory. We performed this small-scale phase II clinical study to investigate the clinical efficacy of anti-PD-1 plus CIK cell therapy in patients with mRCC refractory to previous targeted therapy.

## Materials and Methods

### Study Design and Patients

The inclusion criteria were as follows: i) pathologically confirmed clear cell RCC (ccRCC, the clear cell carcinoma component was seen in the pathological tissue); ii) postoperative recurrence or inoperable resection; iii) failure after at least one type of anti-VEGFR targeted therapy; iv) at least one measurable lesion per Response Evaluation Criteria in Solid Tumors (RECIST) guidelines; v) expected lifespan >3 months; vi) Eastern Cooperative Oncology Group (ECOG) performance status ≤2; vii) aged between 18 and 80 years regardless of sex; viii) <1.5-fold normal serum bilirubin and creatinine levels; and ix) free from chronic active hepatitis, HIV infection, or concomitant corticosteroid or immunosuppressant therapy.

This one-arm, single-center, open clinical study was registered in the Chinese Clinical Trial Registry (ChiCTR-1900024385) upon approval by the ethical committee of the Henan Cancer Hospital (no. 2016119). This study adhered to the tenets of the Declaration of Helsinki. Written informed consent was obtained from all patients for their participation and publication of the results. Between May 2016 and July 2019, 29 patients were enrolled in the study. The median follow-up duration was 37.8 months [95% confidence interval (CI): 32.2–43.6]. Eligible patients received autologous CIK cells (~5 × 10^9^) plus anti-PD-1 (3 mg/kg nivolumab or 2 mg/kg pembrolizumab) administered intravenously every 3 weeks. CIK cell therapy was administered for up to eight cycles and anti-PD-1 antibodies were administered until progressive disease, intolerable adverse events, CR, or at the will of the patients. Tumors were assessed at baseline and every 9 weeks, or when patients experienced discomfort potentially owing to the disease.

### CIK Cell Preparation and Phenotype Analysis

Autologous CIK cells were prepared as described previously (11, 18). Briefly, heparinized peripheral blood (50 ml) was collected 1 week after administration of the anti-PD-1 agent. Peripheral blood mononuclear cells were cultured in GT-T551 medium containing anti-CD3 monoclonal antibody, interferon-γ, interleukin (IL)-2, and RetroNectin. Approximately 5 × 10^9^ cells were harvested and examined for the presence of biological contaminants after 10–14 days and were resuspended in 2% albumin-containing physiological saline solution before transfusion back into the patients. The cells were stained for anti-CD3-APC, anti-CD4-PerCP, anti-CD8-PerCP, and anti-CD56-PE (BD Biosciences, NY, USA) for 20 min on ice. Each dose of the harvested CIK cells contained approximately 5 × 10^9^ cells. All products were free of bacterial and fungal contamination. The endotoxin level was less than five endotoxin units per milliliter.

### Outcomes

The primary endpoint was ORR per RECIST version 1.1, defined as the proportion of patients who achieved a complete or partial response as their best overall response, and the secondary endpoints were progression-free survival (PFS) and overall survival (OS). PFS was defined as the time from the first day of combined therapy to the day of objective tumor progression or death from any cause, whichever occurred first, or if the data were censored upon the final follow-up. OS was defined as the time from the first day of combined therapy to death from any cause, or the censor of data on the final follow-up. The treatment-related adverse events (AEs) were evaluated with respect to incidence and severity according to the National Cancer Institute Common Terminology Criteria for Adverse Events (NCI-CTCAE) version 4.0.

### Statistical Analysis

Statistical analyses were conducted using SPSS Statistics ver. 22.0 (IBM Corp., Armonk, NY, USA). Sample size was calculated by PASS ver. 15.0. The ORR of anti-PD-1 was approximately 20% as second- or later-line therapy for mRCC; thus, it was speculated that combined application of CIK cells and anti-PD-1 agent could increase the ORR to 40%. With a one-sided *α* ≤0.05 as the test level, a sample size of 29 cases was needed when *β* = 0.2. Descriptive statistics were used to summarize the characteristics of the patients, treatment-related AEs, and overall responses. Kaplan–Meier and log-rank tests were performed for survival analysis and the association with potential prognostic factors. The Cox proportional hazards model was used for multivariate analyses through the forward-likelihood ratio method using a significance threshold of 0.1 for the variables. A *p*-value <0.05 obtained by two-sided tests was indicative of statistical significance.

## Results

### Patient Characteristics

**Figure 1:**
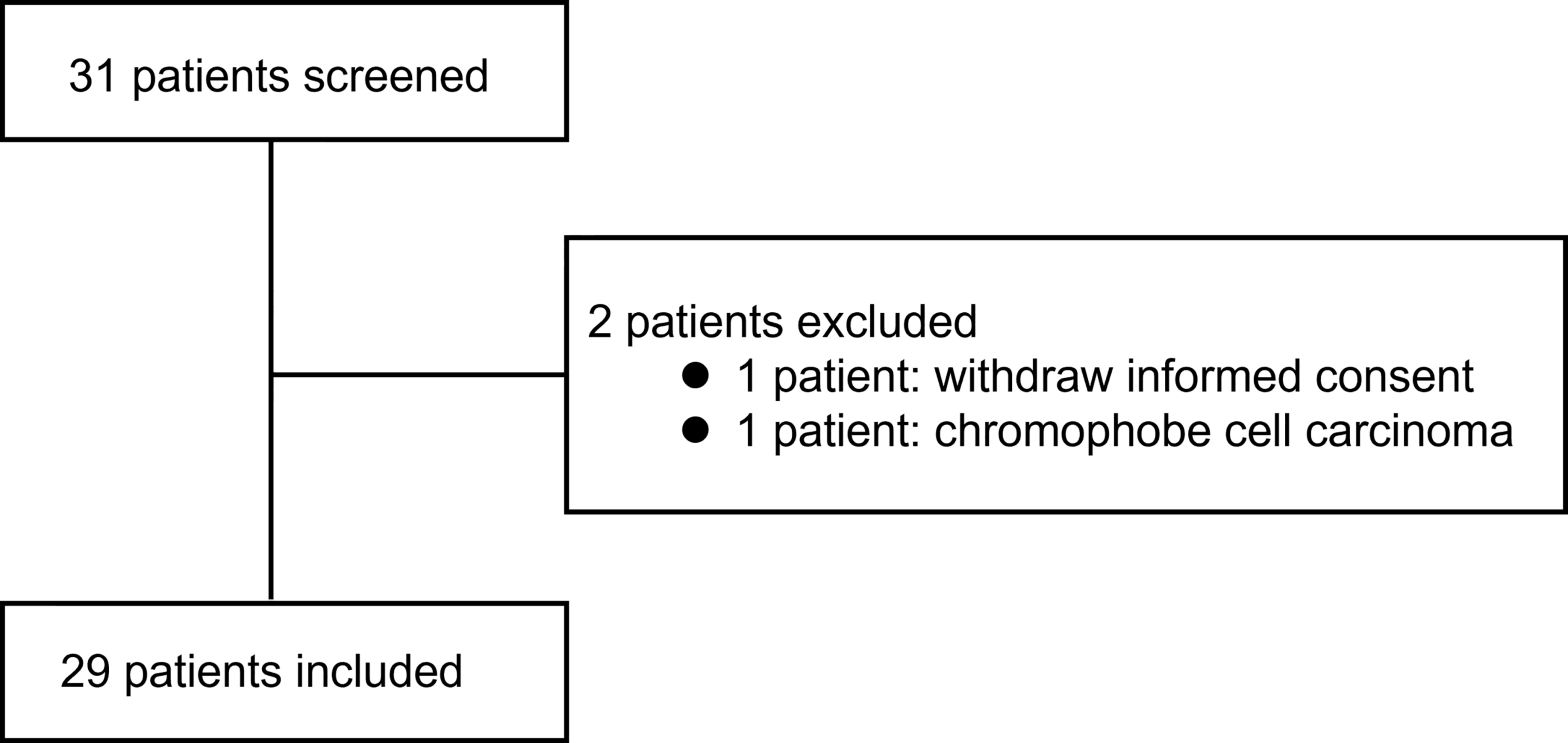
Flowchart of the screening and study inclusion.

**Figure 1** shows the screening process and how patients were included into the analysis. **Table 1** summarizes the patient characteristics.

**Table 1:** Baseline demographic and clinical characteristics of the patients.

Characteristics	Number (%)
Sex	
Male	19 (65.5%)
Female	10 (34.5%)
IMDC risk category	
Favorable	4 (13.8%)
Intermediate	17 (58.6%)
Poor	8 (27.6%)
ECOG PS	
0	4 (13.8%)
1	18 (62.1%)
2	7 (24.1%)
Prior nephrectomy	26 (89.7%)
Prior radiotherapy	6 (20.7%)
Prior systemic therapy	
VEGFR inhibitor	29 (100%)
mTOR inhibitor	3 (10.3%)
Cytokine	10 (34.5%)
Number of prior therapies	
1	15 (51.7%)
2	14 (48.3%)
Metastatic sites	
Liver	8 (27.6%)
Brain	6 (20.7%)
Bone	12 (41.4%)
Lung and pleura	25 (86.2%)
Others^a^	20 (69.0%)

ECOG PS, Eastern Cooperative Oncology Group performance scale; IMDC, International Metastatic Renal-Cell Carcinoma Database Consortium; mTOR, mammalian target of rapamycin; VEGFR, vascular endothelial growth factor receptor.

a Others include subcutaneous nodules, lymph nodes, adrenal gland, pancreas, chest wall, abdominal wall, and intramuscular sites.

### Numbers and Phenotype of CIK Cells

The median count of CIK cells per cycle was 6.4 × 10^9^ (range, 4.8–12 × 10^9^), and the cellular viability reached 90% or higher based on trypan blue staining. Phenotypic analysis of autologous CIK cells before transfusion back to patients showed that the percentages of CD3^+^, CD3^+^CD4^+^, CD3^+^CD8^+^, CD3^+^CD56^+^, and CD3^+^CD56^+^ cell subsets were 96.8% ± 3%, 36.7% ± 14.8%, 59.4% ± 14.6%, 6.5% ± 5.5%, and 1.3% ± 1.0%, respectively.

### Clinical Efficacy

**Figure 2:**
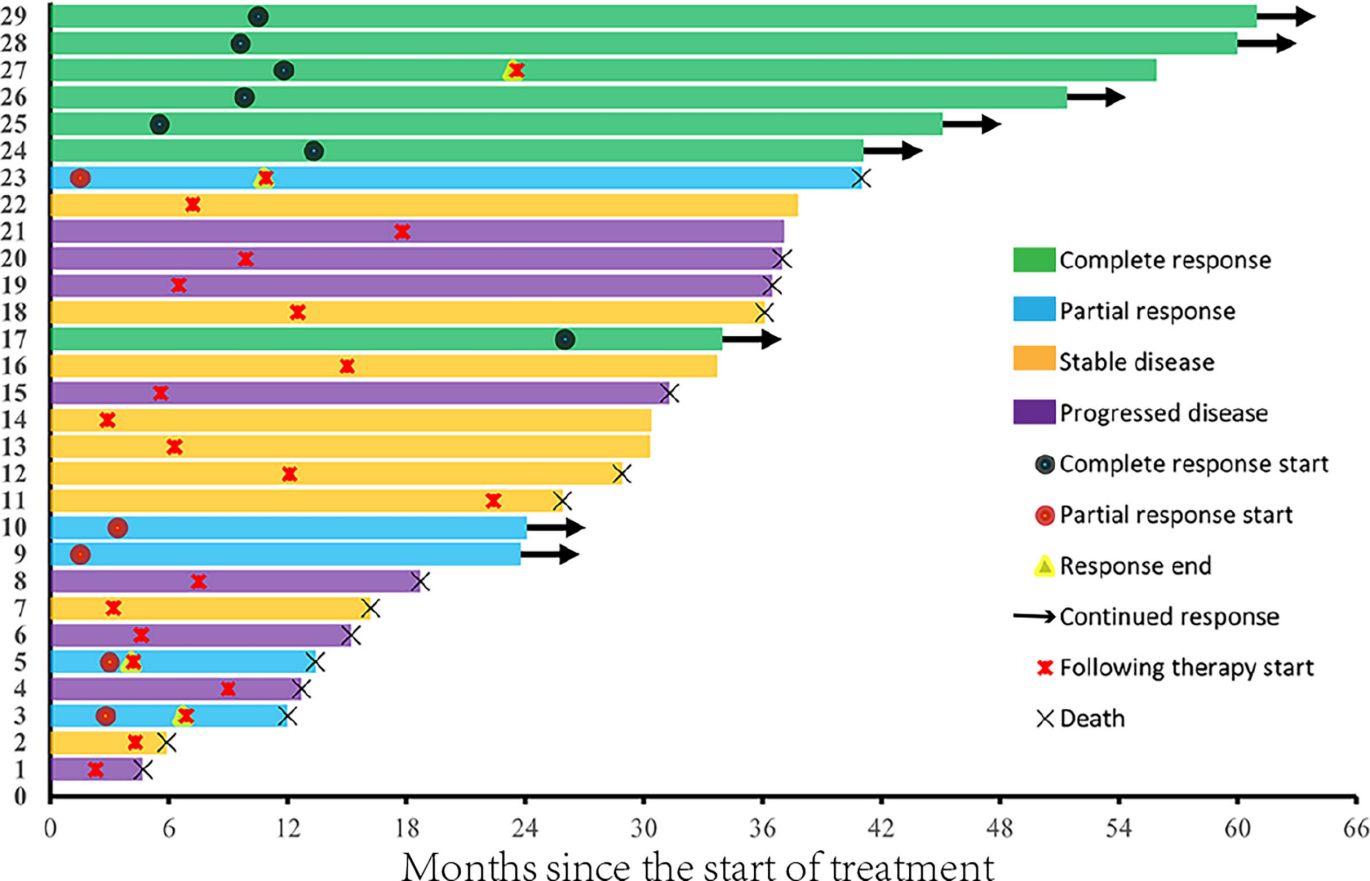
Response to treatment of all study participants (*N* = 29).

The ORR was 41.4% and the CR rate was 24.1%, including patients with progressive disease (PD; *n* = 9), stable disease (SD; *n* = 8), partial response (PR; *n* = 5), and CR (*n* = 7). The CR and PR rates were higher among men than among women (*χ* ^2^ = 6.651, *p* = 0.01). Ten patients received pembrolizumab plus CIK cell therapy and 19 patients received nivolumab plus CIK cell therapy, among which two and five patients achieved CR, respectively (*χ* ^2^ = 0.425, *p* = 0.515). Among the 10 patients who had previously received cytokines, three achieved a CR, one achieved a PR, two had SD, and four experienced PD. Among patients who achieved PR or CR, the median time to the best response was 5.5 months (**Figure 2**). The median treatment duration was 6.1 months. Currently, only one patient is still receiving treatment.

**Figure 3:**
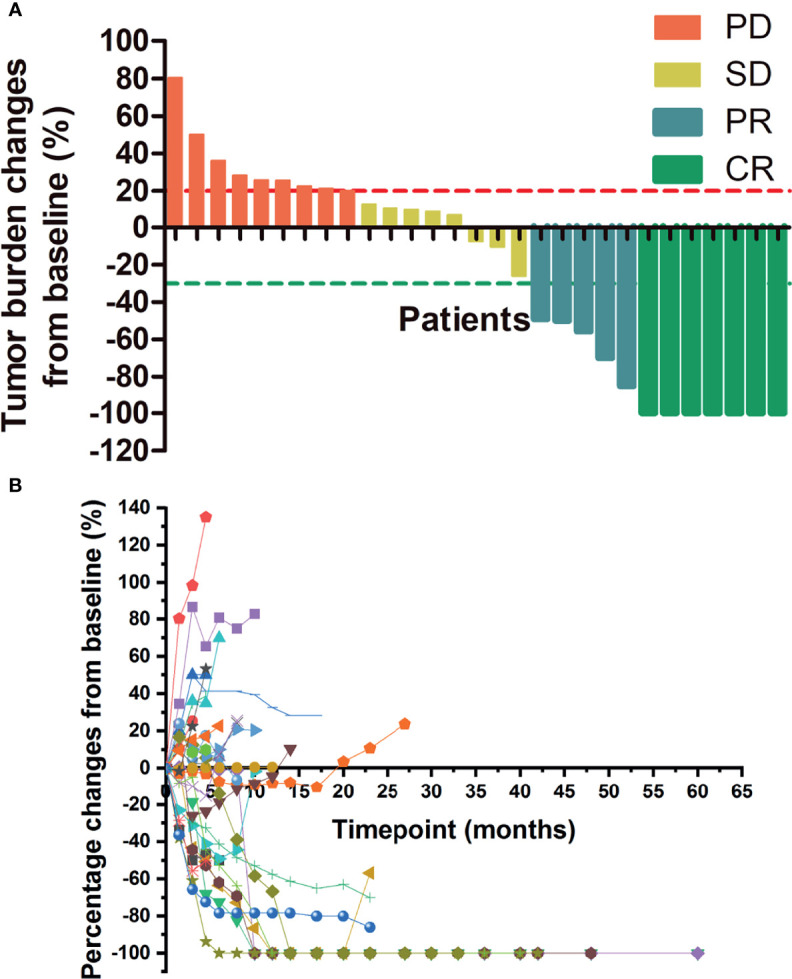
Changes in tumor burden upon treatment initiation. **(A)** Tumor burden changes by best response. **(B)** Percentage changes in lesion size with time. PD, progressive disease; SD, stable disease; CR, complete response; PR, partial response.

**Figure 4:**
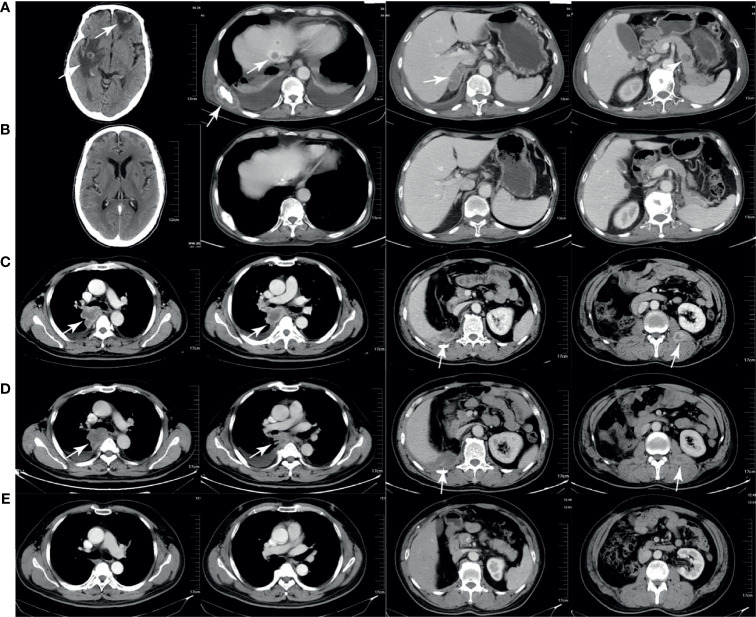
Computed tomography (CT) imaging of two patients with complete response. **(A, B)** CT scans of one patient before and after treatment. The brain, liver, bone, adrenal gland, and pancreas metastases **(A)** disappeared after treatment **(B)**. **(C–E)** CT scans of another patient before treatment, during pseudoprogression, and after treatment. The lung, liver, and intramuscular metastases **(C)** enlarged after 2 months of therapy **(D)** and disappeared after 6 months of therapy **(E)**.

**Figure 3A** shows the tumor burden changes according to the best response and **Figure 3B** shows the tumor burden changes during treatment. **Figure 4** shows the imaging changes of two patients with CR. Twelve patients received continuous treatment upon the initial PD evaluation, among which two, two, three, and five patients had CR, PR, SD, and PD, respectively.

**Figure 5:**
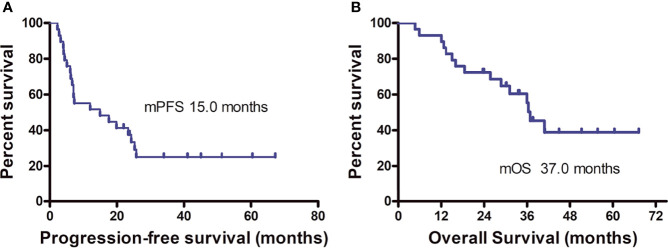
Progression-free survival **(A)** and overall survival **(B)** curves of the patients.

On the final follow-up in June 2021, 21 patients experienced PD and eight were in remission; 15 patients died from disease progression and 14 remained alive. The median PFS was 15.0 months (95% CI: 9.2–33.1 months) and the median OS was 37.0 months (95% CI: 31.0–43.0 months) (**Figure 5**). Among the 12 patients who achieved PR or CR, only four experienced disease progression and the median PFS was not achieved. Univariate analysis revealed that the best therapeutic efficacy alone influenced the median PFS, which was longer in patients with CR and PR than for those with SD and PD (*p* < 0.001). The median PFS of patients that showed SD and PD was 7.1 months (95% CI: 5.8–8.4 months); however, for patients with CR and PR, the median PFS was not available (on data analysis, only four of the 12 patients with CR or PR experienced disease progression). It should be noted that one patient showing CR developed cerebellar metastases 18.8 months after treatment discontinuation. This patient received gamma knife treatment followed by 1-month axitinib treatment. On the final follow-up by the end of June 2021, this patient was still in CR. The detailed information of the patients is listed in **Table 2**.

**Table 2:** Detailed efficacy data of the patients enrolled.

Gender	Age	IMDC category	Metastasis site	Previous VEGF-TKI	Best response	PFS (months)	OS (months)	Status
Male	67	Poor	Bone, liver, brain, adrenal gland, pancreas, pleura	Cytokine–sorafenib–sunitinib	CR	67.4 continued	67.4	Alive
Male	59	Intermediate	Lung	Cytokine–sorafenib	CR	23.4	55.9	Alive
Male	47	Favorable	Pleura	Cytokine–sorafenib	CR	34 continued	34	Alive
Male	79	Poor	Bone, lung, pleura	Axitinib	CR	60.6 continued	60.6	Alive
Male	50	Poor	Bone, lung, chest wall	Sorafenib–axitinib	CR	51.4 continued	54.4	Alive
Male	54	Intermediate	lung, contralateral kidney	Sunitinib	CR	45.1 continued	45.1	Alive
Male	46	Intermediate	Lung, liver, nose, psoas major	Sunitinib	CR	41.1 continued	41.1	Alive
Male	56	Intermediate	Lung	Sorafenib	PR	4.1	13.4	Dead
Male	69	Intermediate	Brain, retroperitoneal lymph nodes	Cytokine–sorafenib–sunitinib	PR	22 continued	24.1	Alive
Male	50	Intermediate	Lung, bone, brain	Sunitinib–everolimus	PR	6.7	12	Dead
Male	48	Intermediate	Lung, brain, adrenal glands	Sorafenib	PR	25.3	41	Dead
Female	65	Intermediate	Lung, mediastinal lymph nodes, intramuscular nodules in chest wall	Sorafenib–everolimus	PR	23.8 continued	23.8	Alive
Female	72	Intermediate	Liver, adrenal gland, pancreas, bone, abdominal wall	Cytokine–sunitinib	SD	24.3	25.9	Dead
Male	54	Intermediate	Lung, liver, adrenal gland, pancreas, bone	Cytokine–sorafenib–axitinib	SD	25.8	36.1	Dead
Female	38	Favorable	Lung, breast, multiple lymph nodes	Sorafenib	SD	7.1	37.8	Alive
Female	63	Poor	Liver, lung	Sorafenib–sunitinib	SD	12	28.9	Dead
Female	31	Poor	Lung, adrenal gland, multiple lymph nodes	Axitinib	SD	2.8	30.4	Alive
Male	54	Poor	Adrenal gland, lung, bone, multiple lymph nodes	Axitinib	SD	15	33.7	Alive
Male	62	Favorable	Facial muscles, lung, pancreas, multiple lymph nodes, node behind the prostate	Sorafenib	SD	6.1	30.3	Alive
Female	66	Intermediate	Lungs, bone, multiple lymph nodes	Sorafenib–axitinib	SD	3.2	16.2	Dead
Male	68	Intermediate	Lung, pleura	Cytokine–sunitinib–sorafenib	PD	6.3	36.5	Dead
Female	53	Favorable	Lung, bone, brain	Cytokine–sunitinib–axitinib	PD	17.6	37.1	Alive
Male	52	Intermediate	Lung, pleura, liver, bone	Cytokine–sunitinib–everolimus	PD	7.1	31.3	Dead
Female	53	Intermediate	Lung, operation area	Cytokine–axitinib	PD	5.1	12.7	Dead
Male	61	Poor	Lung, bone, liver, brain, multiple subcutaneous nodules	Sorafenib	PD	2.3	4.7	Dead
Male	54	Poor	Lung, liver, retroperitoneal lymph nodes	Sorafenib	PD	4	5.9	Dead
Male	47	Intermediate	Lung, pleura, bone, multiple lymph nodes, node in front of psoas major	Sunitinib–cabozantinib	PD	19.9	37	Dead
Female	37	Intermediate	Adrenal gland, multiple lymph nodes	Axitinib–sorafenib	PD	7.3	18.7	Dead
Female	41	Intermediate	Operation area, abdominal pelvic wall, lung, psoas muscle, peritoneum	Sunitinib–axitinib	PD	4.4	15.2	Dead

PD, progressive disease; PR, partial response; CR, complete response; SD, stable disease.

As shown in **Table 3**, the occurrence rate of AEs was 86.2%, with most being of grade 1/2. This combined regimen was well-tolerated and discontinued in two patients (6.9%) due to AEs. One patient presented with grade 3 nausea, and after four cycles of combined therapy, SD (tumor burden enlarged) appeared on computed tomography (CT). This patient was then shifted to axitinib therapy and he did not receive glucocorticoid for nausea, but the symptom resolved 1 month after stopping anti-PD-1 therapy. This patient died from disease progression 3 months later. Grade-2 interstitial pneumonia occurred in one patient at the site of irradiation before immunotherapy; this patient had cough and sputum discharge, which were mitigated after 1.5 months of immunotherapy and symptomatic treatment. After 3 months, CR was detected by positron emission tomography–CT scan evaluation.

**Table 3:** Toxicity profile and safety summary.

Adverse events	Grade 1	Grade 2	Grade 3	Grade 4
	Number (%)			
Nausea	–	–	1 (3.4%)	–
Pyrexia	–	2 (6.9%)	–	–
Hypertension	1 (3.4%)	–	–	–
Transient hyperthyroidism	4 (13.8%)	–	–	–
Hypothyroidism	6 (20.7%)	2 (6.9%)	–	–
Leukopenia	2 (6.9%)	3 (10.3%)	–	–
Anemia	8 (27.6%)	3 (10.3%)	–	–
Elevated transaminase	5 (17.2%)	2 (6.9%)	–	–
Elevated lipase	5 (17.2%)	–	–	–
Elevated creatinine	5 (17.2%)	–	–	–
Triglycerides	2 (6.9%)	–	–	–
Elevated total bilirubin	6 (20.7%)	–	–	–
Hypermagnesemia	1 (3.4%)	–	–	–
Elevated creatine kinase isoenzyme	3 (10.3%)	–	–	–
Interstitial pneumonia	1 (3.4%)	1 (3.4%)	–	–

## Discussion

The combination of anti-PD-1 plus CIK cell therapy attained a high CR rate in patients with refractory ccRCC. Among the 29 patients included in the study, seven presented CR (CR rate, 24.1%), which was higher than those reported in a first-line setting (19–24), despite the small size of the patient cohort evaluated. The median PFS in this study was 15.0 months and the median OS was 37 months. The treatment was discontinued in all seven patients who achieved CR, and in six patients, no treatment interval was more than 2 years. Single intracranial metastasis occurred in one patient who achieved CR. To date, the remaining six patients are still in CR. However, three of the five patients who showed PR experienced disease progression. Taken together, CR during therapy may indicate the curative potential of the treatment for advanced ccRCC.

To achieve a high CR rate and ORR, anti-PD-1 plus anti-angiogenesis therapy has been explored in mRCC. In the CheckMate 9ER study, the ORR of nivolumab plus cabozantinib as first-line therapy for mRCC was 54.8% and the CR rate was 9.3%; the median PFS reached up to 17 months (23). The results of the Keynote CLEAR study were even more encouraging, with an ORR of 71%, CR rate of 16.1%, and median PFS of 23.9 months (24). However, the higher ORR and long median PFS in the CheckMate 9ER and Keynote CLEAR studies were at the cost of higher rates of AEs, with grade-3 or higher AE rates of 70.6% and 82.4%, respectively; 19.7% of patients discontinued therapy because of AEs in CheckMate 9ER, whereas dose interruption or discontinuation occurred in 56% of patients in the Keynote CLEAR trial (23, 24). Moreover, most of the patients included in the Keynote CLEAR trial were patients with favorable and intermediate risk factors (91.2%) (24). With respect to drug accessibility and AEs, these regimens could not be performed in Chinese patients, and exploring anti-PD-1 antibody-based combination in patients with mRCC whose diseases failed to respond to the first-line or second-line anti-angiogenic therapy has important clinical significance. Our results showed good clinical efficacy and tolerance of anti-PD-1 plus autologous CIK cell therapy. Seven of the 29 patients achieved CR and only one relapsed after therapy discontinuation, providing such patients with the possibility of long survival and even a durable clinical benefit.

CIK cells are a population of immune effector cells and have activated T cells and natural killer cell properties, which could be expanded *in vitro* (25). CIK cells showed antitumor activity in many types of tumors. In a randomized phase II study, the ORR of CIK cells as first-line therapy for mRCC was 53% and the CR rate was 18% (10). Retrospective data of our center showed that the ORR of CIK cell monotherapy in mRCC patients was 14% (11). CIK cells have non-MHC–restricted antitumor potential (13) and can selectively infiltrate the tumor tissue, forming an inflammatory tumor microenvironment (12). CIK cell therapy has the potential to reduce the postoperative recurrence rate among patients with hepatocellular carcinoma (26), and a synergistic effect of CIK cells combined with chemotherapy was previously reported (27). At the same time, CIK cells could exert an immunomodulatory function by downregulating MDSC (11). Anti-PD-1/PD-L1 therapy is an effective therapy for mRCC, and the therapeutic effect is mediated through the reinvigoration of tumor-specific T-cell immunity. Studies have shown that tumor regression through anti-PD-1/PD-L1 therapy requires pre-existing T cells within the tumor, which are negatively regulated by PD-1/PD-L1-mediated adaptive immune resistance (5–7). Theoretically, there should be a synergistic effect of anti-PD-1 antibody and CIK cells. After re-transfusion into the body, some CIK cells selectively infiltrate the tumor tissue, leading to the generation of a local inflammatory microenvironment. Expansion of activated T cells in the tumor microenvironment may temporarily exacerbate lesions during treatment, leading to the so-called pseudoprogression on immunotherapy administration. In this study, four patients experienced pseudoprogression, two of which were exacerbations of the original lesions, and both attained CR. New lesions were detected while the original lesions shrank in one patient, and the other patient experienced tumor shrinkage after transient enlargement.

PD-L1 expression on the surface of tumor cells in advanced non-small cell lung cancer without driver gene mutations predicts the efficacy of anti-PD-1 therapy, thereby supporting PD-L1 as a biomarker to guide pembrolizumab-based therapy for this disease (28). However, PD-L1 expression in mRCC could not predict the efficacy of anti-PD-1 therapy as both monotherapy and combinatorial therapy. PD-L1 expression can exhibit temporal and spatial variations within a tumor. Several factors influencing PD-L1 expression may prevent it from being an effective predictor of anti-PD-1 efficacy for mRCC. In particular, the tumor tissue used to detect PD-L1 expression may not be sufficiently fresh, PD-L1 expression may be altered by the tumor microenvironment elements so that the detected PD-L1 expression in cancer samples may not represent the actual status during treatment, and no standardized methods are currently available for the detection and interpretation of PD-L1 expression. In this study, PD-L1 expression was detected in only one patient, representing a rate of <1%. This patient achieved CR, and after 4 years of treatment discontinuation, and the patient is currently in a disease-free state.

This study has some limitations. First, the patient cohort was small. This study was initiated by researchers; although CIK cells are free, nivolumab or pembrolizumab must be paid for by the patients themselves. This resulted in slow patient entry and the weak representativeness of the entry patients. Second, this was an open, one-arm study without a control group, and the results were compared with those of the CheckMate 025 trial, which was conducted primarily in European and American patients. Owing to ethnic differences, it could not be ruled out that the anti-PD-1 agent might be more effective in Chinese patients. Lastly, PD-L1 expression was examined in only one patient and no other biomarkers were screened. However, 24.1% of patients in this study achieved CR and treatment was discontinued for over 2 years in five patients; these patients are currently in remission, indicating that combination therapy may provide a potential durable radiographic response for patients with advanced ccRCC refractory to targeted therapies.

In conclusion, anti-PD-1 and CIK cells exhibit synergistic effects in advanced ccRCC. This combinatorial therapy resulted in a high CR rate and showed good tolerance, providing a potential cure for this malignancy. Nonetheless, additional studies on this treatment regimen to explore its efficacy in a larger cohort and to identify biomarkers predictive of its efficacy are warranted.

## Data Availability Statement

The raw data supporting the conclusions of this article will be made available by the authors, without undue reservation.

## Ethics Statement

The studies involving human participants were reviewed and approved by the Henan Cancer Hospital Ethics Committee. The patients/participants provided their written informed consent to participate in this study.

## Author Contributions

QG: conceptualization, project administration, and supervision. LZ: resources, writing—original draft, writing—review and editing, visualization, and investigation. TL: methodology, formal analysis, writing—original draft, visualization, and investigation. YPS: data curation and validation. YY, BM, YZ, YMS, LH, XF, and LL: resources and investigation. BX, QP, and JG: Methodology. HL and XR: data curation and investigation. ZW: data curation, investigation, and supervision. All authors contributed to the article and approved the submitted version.

## Funding

This work was supported by the Industry-University-Research Collaboration of the Health Commission of the Henan Province (No. 182107000027) and the National Natural Science Foundation of China (No. 81902902). The funding agencies had no role in the collection, analysis, and interpretation of data; in the writing of the report; or in the decision to submit the article for publication.

## Conflict of Interest

The authors declare that the research was conducted in the absence of any commercial or financial relationships that could be construed as a potential conflict of interest.

## Publisher’s Note

All claims expressed in this article are solely those of the authors and do not necessarily represent those of their affiliated organizations, or those of the publisher, the editors and the reviewers. Any product that may be evaluated in this article, or claim that may be made by its manufacturer, is not guaranteed or endorsed by the publisher.
